# NO_2_ and PM_2.5_ air pollution co-exposure and temperature effect modification on pre-mature mortality in advanced age: a longitudinal cohort study in China

**DOI:** 10.1186/s12940-022-00901-8

**Published:** 2022-10-13

**Authors:** John S. Ji, Linxin Liu, Junfeng (Jim) Zhang, Haidong Kan, Bin Zhao, Katrin G. Burkart, Yi Zeng

**Affiliations:** 1 Vanke School of Public Health, Tsinghua University, Beijing, China; 2 Nicholas School of the Environment, Duke University, Durham, NC USA; 3 Global Health Institute, Duke University, Durham, NC USA; 4 School of Public Health, Fudan University, Shanghai, China; 5 School of Architecture, Tsinghua University, Beijing, China; 6 Institute of Health Metrics and Evaluation, University of Washington, Seattle, WA USA; 7 Center for Healthy Aging and Development Studies, National School of Development, Peking University, Beijing, China; 8 Center for the Study of Aging and Human Development and Geriatrics Division, School of Medicine, Duke University, Durham, NC USA

**Keywords:** NO_2_, PM_2.5_, Air Pollution, Mortality, Environmental Epidemiology, China, Elderly

## Abstract

**Background:**

There is a discourse on whether air pollution mixture or air pollutant components are causally linked to increased mortality. In particular, there is uncertainty on whether the association of NO_2_ with mortality is independent of fine particulate matter (PM_2.5_). Furthermore, effect modification by temperature on air pollution-related mortality also needs more evidence.

**Methods:**

We used the Chinese Longitudinal Healthy Longevity Study (CLHLS), a prospective cohort with geographical and socio-economic diversity in China. The participants were enrolled in 2008 or 2009 and followed up in 2011-2012, 2014, and 2017-2018. We used remote sensing and ground monitors to measure nitrogen dioxide (NO_2_), fine particulate matter (PM_2.5_) , and temperature. We used the Cox-proportional hazards model to examine the association between component and composite air pollution and all-cause mortality, adjusted for demographic characteristics, lifestyle, geographical attributes, and temperature. We used the restricted cubic spline to visualize the concentration–response curve.

**Results:**

Our study included 11 835 individuals with an average age of ﻿86.9 (SD: 11.4) at baseline. Over 55 606 person-years of follow-up, we observed 8 216 mortality events. The average NO_2_ exposure was 19.1 μg/m^3^ (SD: 14.1); the average PM_2.5_ exposure was 52.8 μg/m^3^ (SD: 15.9). In the single pollutant models, the mortality HRs (95% CI) for 10 μg/m^3^ increase in annual average NO_2_ or PM_2.5_ was 1.114 (1.085, 1.143) and 1.244 (1.221, 1.268), respectively. In the multi-pollutant model co-adjusting for NO_2_ and PM_2.5_, the HR for NO_2_ turned insignificant: 0.978 (0.950, 1.008), but HR for PM_2.5_ was not altered: 1.252 (1.227, 1.279). PM_2.5_ and higher mortality association was robust, regardless of NO_2_. When acccounting for particulate matter, NO_2_ exposure appeared to be harmful in places of colder climates and higher seasonal temperature variation.

**Conclusions:**

We see a robust relationship of PM_2.5_ exposure and premature mortality in advance aged individuals, however, NO_2_ exposure and mortality was only harmful in places of colder climate such as northeast China, indicating evidence of effect modification by temperature. Analysis of NO_2_ without accounting for its collinearity with PM_2.5,_ may lead to overestimation.

**Supplementary Information:**

The online version contains supplementary material available at 10.1186/s12940-022-00901-8.

## Introduction

Nitrogen dioxide (NO_2_) has harmful health effects. Epidemiological studies indicate NO2 is associated with bronchitis in asthmatic children and reduced pulmonary function [1]. NO_2_ is part of the US EPA six Criteria Air Pollutants (along with carbon monoxide, ground-level ozone, particulate matter, sulfur dioxide, and lead) [2] and the WHO Air quality guidelines (along with particulate matter, ozone, sulfur dioxide) [3]. New 2021 WHO Global Air Quality Guidelines (AQGs) recommendations have lower AQG levels based on mortality or cardiovascular mortality studies [4]. The transportation sector mainly drives NO_2_ exposure. In North America and Europe, urban areas have higher NO_2_ despite low PM. Because PM_2.5_ and NO_2_ tend to be co-exposures, there is no agreement on the causal relationship between NO_2_ and health, particularly mortality. Particulate matter is identified as a causal agent for total, including cardiovascular and respiratory mortality, while nitrogen oxide is only suggestive as a causal agent for total mortality, but it is identified as an acute trigger of poor respiratory function and asthma in the WHO AQG. In China, the interplay of NO_2_ and PM_2.5_, along with temperature effect modification, needs further study.

NO_2_ is a highly reactive gas known as oxides of nitrogen or nitrogen oxides (NOx). NO_2_ forms from emissions from automobile exhaust, power plants, and machinery. Exposure to NO_2_ irritates the airways in the human respiratory system. The health effects of short-term and long-term exposure to NO_2_ are studied separately. Acute NO_2_ exposure has been associated with aggravated respiratory diseases, particularly asthma and pulmonary symptoms [5]. Exposure to NO_2_ may contribute to asthma incidences and other respiratory infections. Several epidemiological studies have linked NO_2_ to mortality. A meta-analysis of 23 studies found a pooled effect on mortality was 1.04 (95% CI 1.02–1.06) with an increase of 10 μg/m^3^ in the annual NO_2_ concentration, independent of the effect of PM_2.5_ [6]. A more recent review found associations between NO_2_ and mortality were attenuated upon adjustment for co-pollutants in some studies while not in others [7]. Furthermore, the WHO AQG specified future research needs on air pollution interaction with high and low temperature, or climatic conditions, on health.

Whether NO_2_ is directly responsible for the health effects or is only an indicator of other pollutants, including particulate matter, evidence from more geographic areas is needed to better understand the concentration–response curve and the generalizability of adverse health effects. First, our study aims to assess the relationship between NO_2_ and mortality in diverse climatic regions of China. Second, we aim to determine the dose–response relation under the new WHO guidelines from 10 to 40 μg/m^3^ annual average exposures. Our unique cohort allows us to assess both high and low NO_2_ exposure regions throughout urban and rural areas of the country. Third, heterogeneous exposures also will enable us to create a multi-pollutant model to assess the collinearity and interaction between NO_2_ and PM_2.5_ and how NO_2_ modifies PM_2.5_ effects and vice versa. Lastly, we aim to look for effect modification by demographic variables such as age, gender, socioeconomic factors, and temperature, to find the most vulnerable group to NO_2_ exposure.

## Methods

### Study population

We used the Chinese Longitudinal Healthy Longevity Survey (CLHLS) datasets. It is a longitudinal cohort designed to study healthy longevity. This cohort aims to gather information of the elderly aged 65 and older in 23 provinces of China. The cohort was initially conceived as a survey to study the senior population’s health status, quality of life, socioeconomic characteristics, family, lifestyle, and demographic profile. We overlaid environmental exposure data based on the residential area with remote sensing. Health endpoints include respondents’ health conditions, daily functioning, self-perceptions of health status and quality of life, life satisfaction, mental attitude, and feelings about aging. We used the 2008-2009 wave of Chinese Longitudinal Healthy Longevity Study (CLHLS) with urban and rural coverage in 23 provinces. The participants were enrolled in 2008 or 2009 and followed up to 2018 roughly every two years.

Among the 16 954 participants in the 2008/2009 cohort, we excluded 3109 participants who were lost in the first follow-up or did not have death time records, 267 participants without matched NO_2_ or PM_2.5_, 1611 participants with missing values in covariates, and 132 participants aged younger than 65 years. We finally included 11 835 participants.

The CLHLS study was approved by research ethics committees of Peking University (IRB00001052-13074) and Duke University. Written informed consent was obtained from each respondent.

### Air pollutant exposure assessment

The concentrations of nitrogen dioxide (NO_2_) concentration levels (μg/m^3^) were obtained at an area-level with spatial resolution up to one-kilometer [8]. Land-use regression model corrected for satellite pass time and cloud coverage was directly used for urban areas. For rural areas, NO2 concentrations were adjusted by using ﻿surface NO2 concentrations derived from the Ozone Monitoring Instrument satellite NO_2_ columns. ﻿Model performance differed regionally and the ﻿ coefficient of determination (R^2^) was 0.52 in Asia, approximately matched the global average (0.54) [9, 10].

We calculated PM_2.5_ concentration at an area-level, with baselayer data at 0.01° × 0.01° resolution obtained from the Atmospheric Composition Analysis Group. Exposure assessment techniques utilized monitors at the ground-level for PM_2.5_ between 1998–2020 (V5.GL.02) by combining Aerosol Optical Depth (AOD) retrievals from the NASA MODIS, MISR, and SeaWIFS instruments with the GEOS-Chem chemical transport model, and subsequently calibrating to global ground-based observations using a Geographically Weighted Regression (GWR), as detailed in the reference [11]. We matched the annual exposure of NO_2_ and PM_2.5_ in the year closest to the mortality. We further used the WHO air quality guidelines [4] and the median as the cut-off point to classify NO_2_ and PM_2.5_ into different categories.

### Mortality outcome assessment

The immediate family members of subjects reported the mortality information in the follow-up surveys. We measured the survival time in months from the first interview to the recorded death date or last interview date.

### Covariates measurements

We included baseline characteristics including age, gender, marital status, education, smoking status, drinking status, physical activity, household income, BMI, ﻿ residence, geographical region of residence, and temperature. We classified marital status into two categories: Currently married and living with spouse as “married” and widowed/separated/divorced/Never married/married but not living with spouse as “not married.” We used the schooling year to evaluate education level and further classified the schooling year into three groups: 0 years (without formal education), 1–6 years (with primary education), and > 6 years (with higher education). We divided the regular exercise, smoking, and alcohol drinking status into three categories: “Current”, “Former”, and “Never” (See supplementary methods). We also quantified the current alcohol drinker based on the kind of alcohol and how much they drank per day. We defined those who drank equal or less than 14 g pure alcohol per day for the female or 28 g per day for the male as light drinkers, otherwise heavy drinkers (See supplementary methods). There were four categories for the annual household income (yuan): < 4000, < 10,000, < 20,000, and ≥ 20,000. We calculated BMI as body weight divided by the square of the body height (unit: kg/m2). We used the WHO standard of BMI, which defined a BMI of < 18.5 kg/m2 as underweight, a BMI of ≥ 18.5 to < 25 kg/m2 as normal weight, a BMI of ≥ 25 to < 30 kg/m2 as overweight, and a BMI of ≥ 30 kg/m2 as obese.﻿ We followed the CLHLS residence categories: “Urban” (including “City” and “Town”) and “Rural.” We divided the geographical region on the basis of residential address to account for climate and dietary differences: central China (Henan, Anhui, Jiangxi, Hubei, Hunan provinces), eastern China (Shandong, Shanghai, Jiangsu, Zhejiang, Fujian provinces), northeastern China (Heilongjiang, Jilin, and Liaoning provinces), northern China (Beijing, Tianjin, Hebei, Shanxi, Shaanxi provinces), southern China (Guangdong, Guangxi, and Hainan provinces), and southwestern China (Chongqing and Sichuan province). Daily meteorological data of the weather monitoring stations across China between 2008 to 2018 was obtained from China Meteorological Administration. Each study participant was matched with meteorological data collected from a monitoring station closest to their area. We used the annual average and standard deviation of the daily mean temperature as the two variables in our analyses.

### Statistical analysis

Given the open cohort nature of our cohort with various subjects contributing different person-times to analysis, we decided to use the Cox proportional hazards model to examine the association between long-term NO_2_ exposure and all-cause mortality. We also calculated the ﻿Cumulative Risk Index (CRI) in the two-pollutant model [12]. These models are adjusted for potential confounders or predictors of outcome: age, gender, marital status, education, smoking status, drinking status, physical activity, household income, BMI, ﻿ residence, and geographical region of residence. We tried to avoid adjusting for mediators so that we do not reduce the explanatory power of exposure variables, recognizing that some variables are time-varying. To assess for non-linearity, we used the restricted cubic spline to describe the concentration–response relationship. Possible effect modifiers such as age and gender were tested via interaction terms and stratified analyses where needed. We also added the temperature mean and temperature variability (SD) in the same year of the air pollution in the model as a sensitivity analysis. We used R 4.0.0 to run all the analyses.

## Results

Those excluded due to the missing of NO_2_ data had similar age, gender, marriage, education, smoking, and alcohol drinking characteristics. Our study included 11 835 individuals, totaling 55 606 person-years of follow-up. During this time, we counted 8 216 mortality events. This high mortality is expected given the average age of our study participants of ﻿86.9 (SD: 11.4) years old at baseline. Representative of demographic distributions on gender, we had a slightly higher proportion of female participants (57.0%). A more significant proportion of our study participants lived in rural areas (63.6%). Many of the study participants received no formal education, which is typical for the historical period of their birth years. The majority of the study participants were currently not married or living with a spouse at baseline (including having a decreased partner), were never smokers, and never consumed alcohol regularly.

The average exposure of NO_2_ in the mortality year was 19.1 μg/m^3^ (SD: 14.1), higher in urban, northern, and eastern regions of China. Participants with higher education or income were also more likely to live in places with higher NO_2_. There were no large variations of NO_2_ exposure by different age groups, gender, marriage, exercise, smoking, and alcohol drinking. The average exposure of PM_2.5_ was 52.8 μg/m^3^ (SD: 15.9), which was similar between urban and rural areas (52.9 vs. 52.8), higher in northern, central, and southwestern regions of China. Participants with older age, no formal education, not married, not exercising currently, not heavy smokers, with higher BMI tended to live in higher PM_2.5_ places compared to their counterparts. There was no noticeable difference in PM_2.5_ exposure for different gender, alcohol drinking status, or household income. Among all the participants, 23.7% (*n* = 2807) were exposed to NO_2_ below the WHO recommended AQG level (< 10 μg/m^3^), and 92.7% (*n* = 10 967) lived in places that reached the interim target 1 (< 40 μg/m^3^). However, only 11.9% (*n* = 1410) of the participants had a PM_2.5_ exposure lower than the interim target 1 level (< 35 μg/m^3^) (Table 1).

**Table 1:** Study population exposure levels relative to 2021 WHO air quality guideline levels

**Variables**	**Overall (*N* = 11,835)**	NO_2_ **(μg/m3)**						**PM**_**2.5**_** (μg/m3)**		
		Median (P25, P75)	AQG level:<10(*n* = 2807)	Interim Target 3: [ 10, 20) (*n* = 4922)	Interim Target 2: [ 20, 30) (*n* = 2374)	Interim Target 1: [ 30, 40) (*n* = 864)	[ 40,109] (*n* = 868)	Median (P25, P75)	Below Interim Target 1: PM_2.5_ < 35 (*N* = 1410)	Above Interim Target 1: PM_2.5_ ≥ 35 (*N* = 10,425)
NO_2_ **(μg/m3)**										
Mean (SD)	19.1 (14.1)	/	6.02 (2.45)	14.9 (2.81)	23.9 (2.75)	34.4 (2.98)	56.7 (16.0)	/	10.9 (9.76)	20.2 (14.2)
Median [Min, Max]	16.0 [1.22, 109]	/	6.01 [1.22, 9.99]	14.8 [10.0, 19.99]	23.4 [20.0, 29.9]	34.0 [30.0, 39.9]	50.8 [40.0, 109]	/	8.70 [1.22, 99.0]	17.0 [1.99, 109]
**PM2.5 (μg/m3)**										
Mean (SD)	52.8 (15.9)	/	42.7 (12.4)	52.5 (13.3)	58.0 (14.7)	60.3 (17.3)	66.1 (20.6)	/	29.8 (4.26)	56.0 (14.2)
Median [Min, Max]	51.2 [14.8, 133]	/	41.1 [14.8, 87.1]	52.1 [19.5, 110]	56.9 [18.7, 120]	58.0 [25.1, 122]	61.7 [24.6, 133]	/	30.9 [14.8, 34.9]	53.6 [35.0, 133]
**Age**										
Mean (SD)	86.9 (11.4)	/	86.5 (11.7)	86.5 (11.3)	87.6 (11.2)	87.3 (11.3)	88.2 (12.2)	/	82.1 (11.1)	87.6 (11.3)
Median [Min, Max]	88.0 [65.0, 116]	/	87.0 [65.0, 112]	88.0 [65.0, 116]	89.0 [65.0, 116]	89.0 [65.0, 113]	90.0 [65.0, 114]	/	81.0 [65.0, 112]	89.0 [65.0, 116]
**Gender: n(%)**										
Male	5071 (42.8)	16.25 (10.51, 23.53)	1176 (41.9)	2091 (42.5)	1063 (44.8)	357 (41.3)	384 (44.2)	50.6 (40.5, 62.9)	642 (45.5)	4429 (42.5)
Female	6764 (57.2)	15.86 (10.22, 22.95)	1631 (58.1)	2831 (57.5)	1311 (55.2)	507 (58.7)	484 (55.8)	51.7 (41.6, 63.6)	768 (54.5)	5996 (57.5)
**Education: n(%)**										
0 year	7469 (63.1)	15.7 (10.15, 22.1)	1816 (64.7)	3260 (66.2)	1504 (63.4)	484 (56.0)	405 (46.7)	52.7 (42.4, 64.3)	805 (57.1)	6664 (63.9)
1–6 years	3242 (27.4)	15.73 (10.23, 24.05)	790 (28.1)	1306 (26.5)	638 (26.9)	230 (26.6)	278 (32.0)	48.1 (39.2, 59.18)	460 (32.6)	2782 (26.7)
> 6 years	1124 (9.5)	20.16 (12.18, 33.34)	201 (7.2)	356 (7.2)	232 (9.8)	150 (17.4)	185 (21.3)	50.5 (40.5, 63.2)	145 (10.3)	979 (9.4)
**Marriage: n(%)**										
Married	3709 (31.3)	15.85 (10.69, 23.45)	830 (29.6)	1583 (32.2)	746 (31.4)	275 (31.8)	275 (31.7)	48 (38.4, 60.3)	598 (42.4)	3111 (29.8)
not married	8126 (68.7)	16.07 (10.17, 23.14)	1977 (70.4)	3339 (67.8)	1628 (68.6)	589 (68.2)	593 (68.3)	52.5 (42.7, 64.3)	812 (57.6)	7314 (70.2)
**Regular Exercise: n(%)**										
Current	3162 (26.7)	18 (12.01, 27.6)	566 (20.2)	1253 (25.5)	657 (27.7)	314 (36.3)	372 (42.9)	50.7 (40.23, 62.68)	453 (32.1)	2709 (26.0)
Former	1460 (12.3)	18.28 (11.11, 28.2)	327 (11.6)	479 (9.7)	317 (13.4)	139 (16.1)	198 (22.8)	52.4 (42.3, 64.3)	175 (12.4)	1285 (12.3)
Never	7213 (60.9)	15.02 (9.64, 21.23)	1914 (68.2)	3190 (64.8)	1400 (59.0)	411 (47.6)	298 (34.3)	51.3 (41.3, 63.4)	782 (55.5)	6431 (61.7)
**Smoking: n(%)**										
Never	7846 (66.3)	15.58 (9.87, 22.86)	2000 (71.3)	3228 (65.6)	1498 (63.1)	552 (63.9)	568 (65.4)	50.8 (40.7, 63.18)	1007 (71.4)	6839 (65.6)
Former	1916 (16.2)	18.06 (12.12, 25.99)	336 (12.0)	778 (15.8)	444 (18.7)	183 (21.2)	175 (20.2)	52.65 (42.8, 64.6)	195 (13.8)	1721 (16.5)
Light smoker	1624 (13.7)	16.25 (10.92, 22.97)	347 (12.4)	713 (14.5)	353 (14.9)	104 (12.0)	107 (12.3)	51.9 (42.6, 63.3)	135 (9.6)	1489 (14.3)
Heavy smoker	449 (3.8)	14.37 (9.17, 20.36)	124 (4.4)	203 (4.1)	79 (3.3)	25 (2.9)	18 (2.1)	48 (38.1, 58.6)	73 (5.2)	376 (3.6)
**Drinking: n(%)**										
Never	8174 (69.1)	16.07 (10.27, 23.29)	1947 (69.4)	3374 (68.5)	1627 (68.5)	616 (71.3)	610 (70.3)	51.1 (41.2, 63.5)	977 (69.3)	7197 (69.0)
Former	1660 (14.0)	16.65 (10.94, 24.23)	368 (13.1)	685 (13.9)	348 (14.7)	120 (13.9)	139 (16.0)	51.85 (41.5, 63.2)	195 (13.8)	1465 (14.1)
Light drinker	754 (6.4)	15.51 (10.38, 23.91)	174 (6.2)	311 (6.3)	142 (6.0)	59 (6.8)	68 (7.8)	52.2 (41.62, 63.5)	83 (5.9)	671 (6.4)
Heavy drinker	1247 (10.5)	15.13 (9.89, 21.35)	318 (11.3)	552 (11.2)	257 (10.8)	69 (8.0)	51 (5.9)	50.6 (40.1, 61.7)	155 (11.0)	1092 (10.5)
**Household Income: n(%)**										
< 4000	2741 (23.2)	13.91 (8.97, 19.29)	798 (28.4)	1339 (27.2)	469 (19.8)	90 (10.4)	45 (5.2)	51.5 (40, 64)	395 (28.0)	2346 (22.5)
< 10,000	2655 (22.4)	14.87 (9.23, 20.22)	747 (26.6)	1211 (24.6)	540 (22.7)	106 (12.3)	51 (5.9)	50.6 (40, 63)	339 (24.0)	2316 (22.2)
< 20,000	2653 (22.4)	16.22 (10.62, 23.23)	591 (21.1)	1134 (23.0)	544 (22.9)	218 (25.2)	166 (19.1)	50.9 (41.6, 62.3)	297 (21.1)	2356 (22.6)
≥ 20,000	3786 (32.0)	19.85 (12.49, 31.96)	671 (23.9)	1238 (25.2)	821 (34.6)	450 (52.1)	606 (69.8)	51.6 (42.6, 63.5)	379 (26.9)	3407 (32.7)
**BMI Groups: n(%)**										
< 18.5	3893 (32.9)	14.31 (8.64, 21.18)	1171 (41.7)	1625 (33.0)	658 (27.7)	225 (26.0)	214 (24.7)	49.9 (40.3, 62.2)	546 (38.7)	3347 (32.1)
< 25	6895 (58.3)	16.56 (10.76, 23.97)	1522 (54.2)	2866 (58.2)	1451 (61.1)	539 (62.4)	517 (59.6)	51.5 (41.3, 63.5)	778 (55.2)	6117 (58.7)
< 30	897 (7.6)	19.43 (13.64, 27.99)	100 (3.6)	374 (7.6)	220 (9.3)	86 (10.0)	117 (13.5)	53 (43.5, 66.2)	76 (5.4)	821 (7.9)
≥ 30	150 (1.3)	21.11 (14.2, 29.16)	14 (0.5)	57 (1.2)	45 (1.9)	14 (1.6)	20 (2.3)	55.4 (43.55, 69.75)	10 (0.7)	140 (1.3)
**Residence: n(%)**										
Urban	4304 (36.4)	20.99 (13.86, 35)	532 (19.0)	1493 (30.3)	924 (38.9)	539 (62.4)	816 (94.0)	50.4 (41.8, 62)	479 (34.0)	3825 (36.7)
Rural	7531 (63.6)	14.04 (8.79, 19.8)	2275 (81.0)	3429 (69.7)	1450 (61.1)	325 (37.6)	52 (6.0)	51.7 (40.8, 63.8)	931 (66.0)	6600 (63.3)
**Geographical Region: n(%)**										
central	2974 (25.1)	15.16 (10.58, 20.13)	657 (23.4)	1555 (31.6)	578 (24.3)	127 (14.7)	57 (6.6)	62.5 (52.3, 70.4)	106 (7.5)	2868 (27.5)
eastern	3387 (28.6)	19.6 (14.36, 26.32)	307 (10.9)	1454 (29.5)	974 (41.0)	350 (40.5)	302 (34.8)	49.3 (41.3, 57.6)	341 (24.2)	3046 (29.2)
northeastern	905 (7.6)	25.16 (17.36, 35.14)	63 (2.2)	246 (5.0)	262 (11.0)	173 (20.0)	161 (18.5)	47.9 (39.2, 57.1)	163 (11.6)	742 (7.1)
northern	648 (5.5)	31.96 (20.15, 50.76)	29 (1.0)	133 (2.7)	140 (5.9)	90 (10.4)	256 (29.5)	73.45 (57.5, 94.43)	19 (1.3)	629 (6.0)
southern	2390 (20.2)	8.84 (4.02, 13.58)	1342 (47.8)	809 (16.4)	154 (6.5)	61 (7.1)	24 (2.8)	40.3 (33.7, 46.5)	732 (51.9)	1658 (15.9)
southwestern	1531 (12.9)	14.73 (9.6, 20.26)	409 (14.6)	725 (14.7)	266 (11.2)	63 (7.3)	68 (7.8)	57.4 (45.6, 67.1)	49 (3.5)	1482 (14.2)
**Temperature mean of the last year (﻿°C)**										
Mean (SD)	16.4 (4.19)	/	19.1 (3.48)	16.3 (3.71)	14.7 (3.75)	14.5 (4.29)	12.4 (4.79)	/	18.7 (5.94)	16.0 (3.74)
Median [Min, Max]	16.6 [0.132, 25.3]	/	19.0 [0.132, 25.3]	16.2 [0.459, 24.7]	15.0 [0.590, 24.7]	15.3 [2.79, 23.6]	14.0 [3.42, 23.6]	/	20.6 [0.132, 25.3]	16.2 [1.98, 24.0]
Missing	1076 (9.1%)	/	103 (3.7%)	358 (7.3%)	158 (6.7%)	78 (9.0%)	379 (43.7%)	/	43 (3.0%)	1033 (9.9%)
**Temperature SD of the last year (﻿°C)**										
Mean (SD)	9.10 (2.13)	/	7.71 (1.76)	9.14 (1.82)	9.92 (1.98)	10.1 (2.18)	11.1 (2.71)	/	7.84 (3.00)	9.29 (1.91)
Median [Min, Max]	9.26 [4.28, 17.2]	/	7.64 [4.28, 17.1]	9.41 [4.28, 17.2]	9.87 [4.33, 17.1]	9.81 [5.90, 16.9]	10.5 [5.94, 16.6]	/	6.98 [4.28, 17.2]	9.40 [5.03, 17.1]
Missing	1076 (9.1%)	/	103 (3.7%)	358 (7.3%)	158 (6.7%)	78 (9.0%)	379 (43.7%)	/	43 (3.0%)	1033 (9.9%)

The Pearson correlation coefficient between NO_2_ (μg/m^3^) and PM_2.5_ (μg/m^3^) was 0.37 (95% CI: 0.35, 0.38). To look for a contrast between NO_2_ and PM_2.5_, we looked for concordance and discordance statistics using the 16 μg/m^3^ for NO_2_ and 51 μg/m^3^ for PM_2.5_ as cut-off points, indicative of median concentrations. There were 17.0% (*n* = 2007) living in places with high NO_2_ and low PM_2.5_, 17.7% (*n* = 2094) living under low NO_2_ and high PM_2.5_, 32.3% (*n* = 3820) living under low NO_2_ and low PM_2.5_, and 33.1% (*n* = 3914) living under high NO_2_ and high PM_2.5_. Additionally, the annual average NO_2_ and PM_2.5_ were both significantly negatively associated with the annual average temperature [Pearson coefficient (95%CI): -0.44 (-0.45, -0.42) and -0.30 (-0.32, -0.28) respectively], and significantly positively associated with annual temperature SD [Pearson coefficient (95%CI): 0.44 (0.42, 0.45) and 0.31 (0.30, 0.33) respectively].

As expected in the single pollutant model, higher NO_2_ was associated with a greater risk for mortality, with the hazard ratio (HR, 95% CI) of 1.114 (1.085, 1.143) for per 10 μg/m^3^ increase after adjusting for demographics, lifestyles, living regions, BMI, annual average temperature, and annual temperature SD. However, after adjusting for PM_2.5_, the association between NO_2_ and mortality was reversed but not significant [HR (95% CI) for per 10 μg/m^3^ increase: 0.978 (0.950, 1.008). Higher PM_2.5_ was consistently associated with higher mortality risk [HR (95% CI) for per 10 μg/m^3^ increase in the single pollutant and two-pollutant model: 1.244 (1.221, 1.268) vs. 1.252 (1.227, 1.279)] (Tables 2 and 3). As we can see, after adjusting for annual average temperature and annual temperature SD in the single pollutant model, the association between NO_2_ and mortality became stronger while there was no significant change for PM_2.5_. We calculated the ﻿HR (95% CI) for cumulative risk estimates from the two-pollutant model as 1.23824 [1.23823, 1.23825].

**Table 2:** Association between NO_2_, PM_2.5_ concentrations and all-cause mortality by model saturation

Adjustment variables	Single pollutant model-NO_2_ (10 μg/m3 increment)		Single pollutant model-PM_2.5_ (10 μg/m3 increment)	
	**HR (95% CI)**	***p* value**	**HR (95% CI)**	***p* value**
Age, gender	1.040 (1.024, 1.056)	< 0.001	1.160 (1.145, 1.175)	< 0.001
Age, gender, education, household income	1.050 (1.033, 1.067)	< 0.001	1.161 (1.146, 1.177)	< 0.001
Age, gender, education, household income, marital status, smoking status, drinking status, physical activity, residence	1.065 (1.047, 1.084)	< 0.001	1.161 (1.145, 1.176)	< 0.001
Age, gender, education, household income, marital status, smoking status, drinking status, physical activity, residence, geographical region of residence	1.072 (1.052, 1.093)	< 0.001	1.245 (1.224, 1.267)	< 0.001
Age, gender, education, household income, marital status, smoking status, drinking status, physical activity, ﻿residence, geographical region of residence, BMI	1.074 (1.053, 1.095)	< 0.001	1.249 (1.228, 1.271)	< 0.001
Age, gender, education, household income, marital status, smoking status, drinking status, physical activity, ﻿residence, geographical region of residence, BMI, annual temperature mean, annual temperature standard deviation	1.114 (1.085, 1.143)	< 0.001	1.244 (1.221, 1.268)	< 0.001

**Table 3:** HR (95% CI) for all-cause mortality considering both NO_2_ and PM_2.5_

**Model**	**NO**_**2**_** or PM**_**2.5**_** (μg/m3)**	**n**	**Two pollutants model—**NO_2_** + PM**_**2.5**_	
			**HR (95% CI)**	***p* value**
**Model a**	**Per 10 μg/m3 increment**			
	**NO**_**2**_	10,759	0.978 (0.950, 1.008)	0.148
	**PM**_**2.5**_	10,759	1.252 (1.227, 1.279)	< 0.001
**Model b**	**NO**_**2**_** (μg/m3)**			
	[ 1.22, 10.00)	2704	1.111 (0.965, 1.278)	0.143
	[ 10.00, 20.00)	4564	0.980 (0.861, 1.114)	0.753
	[ 20.00, 30.00)	2216	1.101 (0.970, 1.250)	0.136
	[ 30.00, 40.00)	786	1.019 (0.884, 1.174)	0.795
	[ 40.00,109.04]	489	Reference	/
	**PM**_**2.5**_** (μg/m3)**			
	[ 14.8, 25.0)	218	0.171 (0.134, 0.218)	< 0.001
	[ 25.0, 35.0)	1149	0.209 (0.183, 0.237)	< 0.001
	[ 35.0, 50.0)	3834	0.516 (0.473, 0.563)	< 0.001
	[ 50.0, 70.0)	4226	0.728 (0.677, 0.783)	< 0.001
	[ 70.0,133.1]	1332	Reference	/
**Model c**	**Combination of NO**_**2**_** and PM**_**2.5**_** (median cut-off)**			
	NO_2_ < 16 & PM2.5 < 51	3701	Reference	/
	NO_2_ ≥ 16 & PM2.5 < 51	1765	1.295 (1.197, 1.401)	< 0.001
	NO_2_ < 16 & PM2.5 ≥ 51	1889	1.641 (1.521, 1.770)	< 0.001
	NO_2_ ≥ 16 & PM2.5 ≥ 51	3404	1.843 (1.715, 1.981)	< 0.001
**Model d**	**Combination of** NO_2_ **and PM**_**2.5**_** (guideline cut-off)**			
	NO_2_ < 20 & PM_2.5_ < 35	1221	Reference	/
	NO_2_ ≥ 20 & PM_2.5_ < 35	146	1.797 (1.380, 2.338)	< 0.001
	NO_2_ < 20 & PM_2.5_ ≥ 35	6047	2.756 (2.501, 3.038)	< 0.001
	NO_2_ ≥ 20 & PM_2.5_ ≥ 35	3345	3.241 (2.909, 3.610)	< 0.001

Model a, b, c, and d all adjusted for age, gender, education, household income, marital status, smoking status, drinking status, physical activity, residence, geographical region of residence, BMI, annual temperature mean, and annual temperature standard deviation

There was a significant negative interaction between NO_2_ and PM_2.5_ (﻿*β*_interaction_ = -0.06, *P*_interaction_ < 0.001), and higher NO_2_ was associated with higher mortality risk only when PM_2.5_ was lower than 53.3 μg/m^3^.

**Fig. 1:**
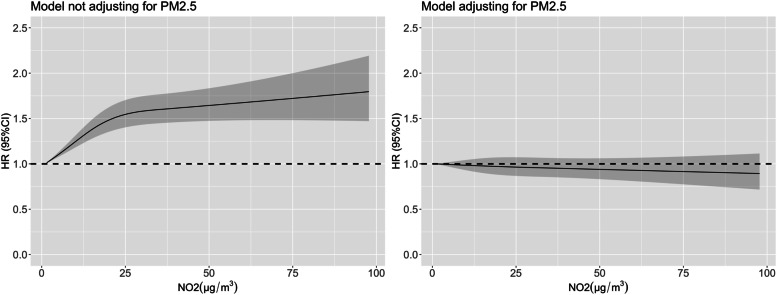
Restricted cubic spline describing effect of the NO_2_ of the year closest to death on mortality before and after adjusting for PM_2.5_. Note: The left figure adjusted for age, gender, education, household income, marital status, smoking status, drinking status, physical activity, residence, geographical region of residence, and BMI. The right figure additionally adjusted for PM_2.5_

The restricted cubic spline for NO_2_ was supralinear, which means there were larger changes in risk for low concentrations compared with higher concentrations. Meanwhile, the spline also showed a reverse before and after adjusting for PM_2.5_ (Fig. 1).

The association between NO2 and mortality remained significantly positive after adjusting for PM_2.5_ in some subgroups. It was even stronger than PM_2.5_ for those living in northeastern China [HR (95% CI: 1.125 (1.035, 1.224) vs. 0.943 (0.858, 1.035)]. NO_2_ was also still positively associated with mortality after adjusting for PM_2.5_ in areas with relatively high NO_2_, low annual temperature mean, or high annual temperature SD [HR (95% CI: 1.047 (1.003, 1.093), 1.083 (1.031, 1.138), and 1.134 (1.08, 1.191) respectively], but negatively associated with mortality in areas with low NO_2_, high annual temperature mean, or low annual temperature SD [HR (95% CI: 0.749 (0.681, 0.823), 0.843 (0.793, 0.896), and 0.876 (0.833, 0.922) respectively] (Table 4). Perhaps, the NO_2_ harm effect is more pronounced in colder regions, with mechanisms to be explored in future studies. The spline stratified by exposure level also showed that NO_2_ tended to have a harmful effect in areas with high NO_2_ (Figure S2). We found those living under exposure to high NO_2_ and low PM_2.5_ were mostly in relatively prosperous regions near the Yangtze Delta (Shanghai, Zhejiang, Jiangsu), while those under the exposure of high NO_2_ and high PM_2.5_ were more likely to be in the northern Jing-jin-ji (Beijing, Tianjin, Hebei) areas with a higher concentration of heavy industry.

**Table 4:** The association between NO_2_, PM_2.5_ and mortality in subgroups

Subgroups	N (%)	Single pollutant model				Two-pollutant model			
		**NO**_**2**_** (per 10 μg/m3)**		**PM**_**2.5**_** (per 10 μg/m3)**		**NO**_**2**_** (per 10 μg/m3)**		**PM**_**2.5**_** (per 10 μg/m3)**	
		**HR (95% CI)**	***p* value**	**HR (95% CI)**	***p* value**	**HR (95% CI)**	***p* value**	**HR (95% CI)**	***p* value**
Age < 75	2077 (19.3%)	1.301 (1.179, 1.434)	< 0.001	1.824 (1.697, 1.96)	< 0.001	0.845 (0.75, 0.953)	0.006	1.93 (1.777, 2.096)	< 0.001
Age: [75, 85)	2282 (21.2%)	1.298 (1.215, 1.387)	< 0.001	1.538 (1.471, 1.609)	< 0.001	0.967 (0.894, 1.046)	0.402	1.554 (1.477, 1.635)	< 0.001
Age: [85, 95)	3410 (31.7%)	1.105 (1.057, 1.155)	< 0.001	1.231 (1.193, 1.27)	< 0.001	0.969 (0.921, 1.019)	0.218	1.243 (1.2, 1.287)	< 0.001
Age ≥ 95	2990 (27.8%)	1.02 (0.978, 1.065)	0.357	1.059 (1.026, 1.093)	< 0.001	0.99 (0.945, 1.037)	0.674	1.062 (1.026, 1.099)	0.001
Female	6127 (56.9%)	1.119 (1.081, 1.158)	< 0.001	1.225 (1.195, 1.256)	< 0.001	1.001 (0.963, 1.041)	0.954	1.225 (1.191, 1.259)	< 0.001
Male	4632 (43.1%)	1.113 (1.068, 1.16)	< 0.001	1.276 (1.24, 1.314)	< 0.001	0.947 (0.903, 0.992)	0.022	1.298 (1.257, 1.34)	< 0.001
Urban	3746 (34.8%)	1.057 (1.018, 1.096)	0.004	1.18 (1.142, 1.219)	< 0.001	0.962 (0.922, 1.003)	0.071	1.199 (1.155, 1.244)	< 0.001
Rural	7013 (65.2%)	1.219 (1.172, 1.268)	< 0.001	1.295 (1.264, 1.326)	< 0.001	1.04 (0.996, 1.086)	0.079	1.284 (1.251, 1.317)	< 0.001
Education:0 year	6868 (63.8%)	1.102 (1.067, 1.138)	< 0.001	1.214 (1.187, 1.243)	< 0.001	0.986 (0.952, 1.022)	0.455	1.219 (1.189, 1.25)	< 0.001
Education:1–6 year	2928 (27.2%)	1.147 (1.088, 1.21)	< 0.001	1.34 (1.289, 1.392)	< 0.001	0.946 (0.89, 1.006)	0.077	1.363 (1.305, 1.423)	< 0.001
Education: > 6 year	963 (9.0%)	1.15 (1.039, 1.273)	0.007	1.241 (1.152, 1.337)	< 0.001	1.026 (0.916, 1.148)	0.661	1.231 (1.135, 1.337)	< 0.001
Smoking: Heavy smoker	413 (3.8%)	1.372 (1.124, 1.675)	0.002	1.571 (1.367, 1.804)	< 0.001	0.898 (0.699, 1.153)	0.398	1.636 (1.384, 1.934)	< 0.001
Smoking: Light smoker	1485 (13.8%)	1.16 (1.076, 1.25)	< 0.001	1.439 (1.356, 1.526)	< 0.001	0.956 (0.88, 1.038)	0.282	1.459 (1.368, 1.556)	< 0.001
Smoking: Former	1731 (16.1%)	1.092 (1.027, 1.161)	0.005	1.191 (1.141, 1.244)	< 0.001	0.981 (0.916, 1.051)	0.594	1.198 (1.142, 1.257)	< 0.001
Smoking: Never	7130 (66.3%)	1.111 (1.075, 1.148)	< 0.001	1.227 (1.198, 1.256)	< 0.001	0.985 (0.949, 1.022)	0.415	1.232 (1.201, 1.264)	< 0.001
Alcohol: Heavy drinker	1127 (10.5%)	1.172 (1.068, 1.287)	0.001	1.397 (1.309, 1.492)	< 0.001	0.966 (0.872, 1.07)	0.504	1.411 (1.314, 1.515)	< 0.001
Alcohol: Moderate drinker	661 (6.1%)	1.115 (0.992, 1.253)	0.068	1.248 (1.141, 1.365)	< 0.001	0.992 (0.87, 1.13)	0.9	1.251 (1.134, 1.381)	< 0.001
Alcohol: Former	1502 (14.0%)	1.011 (0.946, 1.081)	0.742	1.249 (1.187, 1.314)	< 0.001	0.872 (0.809, 0.94)	< 0.001	1.303 (1.233, 1.378)	< 0.001
Alcohol: Never	7469 (69.4%)	1.133 (1.098, 1.17)	< 0.001	1.23 (1.202, 1.258)	< 0.001	1.006 (0.97, 1.042)	0.761	1.228 (1.198, 1.259)	< 0.001
Ability to exercise regularily: current	2712 (25.2%)	1.141 (1.083, 1.202)	< 0.001	1.266 (1.216, 1.318)	< 0.001	1.009 (0.952, 1.07)	0.763	1.262 (1.208, 1.32)	< 0.001
Ability to exercise regularily: Former	1294 (12.0%)	1.003 (0.937, 1.074)	0.933	1.145 (1.09, 1.203)	< 0.001	0.905 (0.837, 0.979)	0.013	1.181 (1.118, 1.247)	< 0.001
Ability to exercise regularily: Never	6753 (62.8%)	1.143 (1.104, 1.184)	< 0.001	1.272 (1.241, 1.303)	< 0.001	0.993 (0.955, 1.033)	0.73	1.274 (1.241, 1.309)	< 0.001
Marriage_status: Married	3399 (31.6%)	1.227 (1.159, 1.3)	< 0.001	1.433 (1.377, 1.492)	< 0.001	0.969 (0.908, 1.035)	0.35	1.447 (1.383, 1.513)	< 0.001
Marriage_status:Not married	7360 (68.4%)	1.083 (1.051, 1.116)	< 0.001	1.194 (1.168, 1.22)	< 0.001	0.978 (0.946, 1.011)	0.189	1.202 (1.173, 1.23)	< 0.001
Household income: < 4000	2634 (24.5%)	1.221 (1.141, 1.308)	< 0.001	1.361 (1.304, 1.421)	< 0.001	1.02 (0.945, 1.1)	0.617	1.355 (1.294, 1.42)	< 0.001
Household income: < 10,000	2472 (23.0%)	1.122 (1.056, 1.194)	< 0.001	1.279 (1.227, 1.333)	< 0.001	0.964 (0.899, 1.033)	0.297	1.291 (1.235, 1.35)	< 0.001
Household income: < 20,000	2393 (22.2%)	1.149 (1.089, 1.212)	< 0.001	1.264 (1.214, 1.315)	< 0.001	1.026 (0.968, 1.088)	0.387	1.255 (1.202, 1.31)	< 0.001
Household income: ≥ 20,000	3260 (30.3%)	1.089 (1.043, 1.138)	< 0.001	1.181 (1.142, 1.221)	< 0.001	0.988 (0.94, 1.037)	0.618	1.186 (1.143, 1.231)	< 0.001
BMI: < 18.5	3628 (33.7%)	1.073 (1.027, 1.121)	0.002	1.23 (1.189, 1.271)	< 0.001	0.964 (0.919, 1.012)	0.137	1.242 (1.198, 1.288)	< 0.001
BMI: < 25	6232 (57.9%)	1.121 (1.081, 1.161)	< 0.001	1.262 (1.231, 1.293)	< 0.001	0.966 (0.928, 1.006)	0.096	1.274 (1.24, 1.31)	< 0.001
BMI: < 30	770 (7.2%)	1.251 (1.129, 1.386)	< 0.001	1.228 (1.14, 1.322)	< 0.001	1.12 (0.994, 1.262)	0.063	1.181 (1.085, 1.285)	< 0.001
BMI: ≥ 30	129 (1.2%)	1.473 (1.042, 2.082)	0.028	1.548 (1.173, 2.044)	0.002	1.195 (0.812, 1.76)	0.367	1.457 (1.071, 1.981)	0.017
Region: central	2974 (27.6%)	1.174 (1.102, 1.251)	< 0.001	1.246 (1.198, 1.296)	< 0.001	0.981 (0.91, 1.056)	0.607	1.253 (1.198, 1.31)	< 0.001
Region: eastern	3147 (29.2%)	1.269 (1.207, 1.335)	< 0.001	1.263 (1.217, 1.311)	< 0.001	1.151 (1.088, 1.217)	< 0.001	1.217 (1.169, 1.267)	< 0.001
Region: northeastern	904 (8.4%)	1.107 (1.022, 1.2)	0.013	0.98 (0.897, 1.071)	0.66	1.125 (1.035, 1.224)	0.006	0.943 (0.858, 1.035)	0.217
Region: northern	354 (3.3%)	1.227 (1.08, 1.394)	0.002	1.114 (1.038, 1.195)	0.003	1.15 (0.986, 1.341)	0.074	1.068 (0.981, 1.162)	0.128
Region: southern	2390 (22.2%)	1.027 (0.959, 1.1)	0.448	2.057 (1.906, 2.219)	< 0.001	0.824 (0.766, 0.886)	< 0.001	2.218 (2.044, 2.407)	< 0.001
Region: southwestern	990 (9.2%)	0.941 (0.866, 1.024)	0.158	1.885 (1.755, 2.025)	< 0.001	0.728 (0.668, 0.794)	< 0.001	2.051 (1.904, 2.21)	< 0.001
Self-reported respiratory disease: No	9718 (90.3%)	1.124 (1.093, 1.156)	< 0.001	1.247 (1.223, 1.273)	< 0.001	0.989 (0.958, 1.02)	0.487	1.251 (1.224, 1.279)	< 0.001
Self-reported respiratory disease: Yes	884 (8.2%)	1.062 (0.971, 1.162)	0.188	1.219 (1.138, 1.305)	< 0.001	0.934 (0.843, 1.035)	0.192	1.247 (1.155, 1.346)	< 0.001
Self-reported cardiovascular disease: No	10,020 (93.1%)	1.118 (1.088, 1.15)	< 0.001	1.253 (1.229, 1.278)	< 0.001	0.98 (0.951, 1.011)	0.208	1.26 (1.233, 1.288)	< 0.001
Self reported cardiovascular disease: Yes	568 (5.3%)	1.033 (0.919, 1.16)	0.589	1.074 (0.988, 1.168)	0.093	0.987 (0.867, 1.123)	0.84	1.078 (0.983, 1.183)	0.108
NO_2_ < 16	5590 (52.0%)	1.036 (0.949, 1.131)	0.432	1.521 (1.47, 1.574)	< 0.001	0.749 (0.681, 0.823)	< 0.001	1.566 (1.512, 1.623)	< 0.001
NO_2_ ≥ 16	5169 (48.0%)	1.098 (1.054, 1.143)	< 0.001	1.11 (1.082, 1.14)	< 0.001	1.047 (1.003, 1.093)	0.037	1.1 (1.07, 1.131)	< 0.001
PM_2.5_ < 51	5466 (50.8%)	1.109 (1.061, 1.158)	< 0.001	2.006 (1.895, 2.124)	< 0.001	0.967 (0.923, 1.013)	0.156	2.026 (1.91, 2.149)	< 0.001
PM_2.5_ ≥ 51	5293 (49.2%)	1.054 (1.016, 1.094)	0.005	1.051 (1.018, 1.085)	0.002	1.038 (0.998, 1.08)	0.062	1.04 (1.005, 1.076)	0.025
Temperature mean: [ 0.132,15.1)	3587 (33.3%)	1.143 (1.095, 1.194)	< 0.001	1.109 (1.077, 1.141)	< 0.001	1.083 (1.031, 1.138)	0.001	1.082 (1.048, 1.118)	< 0.001
Temperature mean: [15.083,17.8)	3587 (33.3%)	1.071 (1.02, 1.124)	0.005	1.254 (1.209, 1.3)	< 0.001	0.96 (0.911, 1.011)	0.118	1.267 (1.219, 1.318)	< 0.001
Temperature mean: [17.822,25.3]	3585 (33.3%)	1.094 (1.034, 1.156)	0.002	2.025 (1.918, 2.137)	< 0.001	0.843 (0.793, 0.896)	< 0.001	2.132 (2.014, 2.257)	< 0.001
Temperature SD: [4.28, 8.17)	3588 (33.3%)	1.036 (0.987, 1.088)	0.151	1.708 (1.635, 1.785)	< 0.001	0.876 (0.833, 0.922)	< 0.001	1.762 (1.684, 1.844)	< 0.001
Temperature SD: [8.17, 9.81)	3585 (33.3%)	1.128 (1.069, 1.19)	< 0.001	1.288 (1.24, 1.338)	< 0.001	0.982 (0.925, 1.042)	0.547	1.294 (1.242, 1.349)	< 0.001
Temperature SD: [9.81,17.18]	3586 (33.3%)	1.227 (1.175, 1.282)	< 0.001	1.174 (1.139, 1.21)	< 0.001	1.134 (1.08, 1.191)	< 0.001	1.128 (1.091, 1.168)	< 0.001

All models adjusted for age, gender, education, household income, marital status, smoking status, drinking status, physical activity, residence, geographical region of residence, BMI, annual temperature mean, annual temperature standard deviation and excluded the adjustment of the subgroup variable

Using the four categorical combination terms of NO_2_ and PM_2.5_ as the independent variable in cox model: those under high NO_2_ and low PM_2.5_ exposure had higher mortality risk than those living in low NO_2_ and low PM_2.5_ areas, and those under high NO_2_ and high PM_2.5_ exposure also had higher risk than those living in low NO_2_ and high PM_2.5_ areas (Table 3s).

## Discussion

In our analysis, we saw that the effect of NO_2_ disappeared or reversed after adjusting for PM_2.5_. Several explanations are possible. First, there may be high collinearity of NO_2_ and PM_2.5_, and the explanatory power of one variable was overshadowed statistically by the other variable. However, we found this explanation unsatisfactory because the effect of PM_2.5_ did not change drastically, and only NO_2_ changed. Second, the phenomenon may manifest the reversal paradox, where the association between an outcome variable and an explanatory (predictor) variable is reversed when another explanatory variable is added to the analysis [13, 14]. This may be a form of Simpson’s Paradox, which refers to a phenomenon whereby the association between a pair of variables (X, Y) reverses sign upon conditioning of a third variable, Z, regardless of the value taken by Z [15]. In our case, the effect estimate direction of NO_2_ reverses when adjusted for PM_2.5_. However, in the two pollutant model, the PM_2.5_ effect was not severely modified by NO_2_, but the presence of a significant interaction suggests a complex relationship. Other descriptions of this phenomenon are termed Lord’s Paradox or suppression effects.

We attempted to examine this relationship visually in Figure S2. PM_2.5_ and NO_2_ may rise and fall together in urban areas, thus making it difficult to separate the effects. To explore possible Simpson’s Bias, we categorized PM_2.5_ and NO_2_ by high and low and looked at the HR within each category. We also quantified the number of participants who fall into the WHO recommended AQG levels and interim targets. We can see that the association between PM_2.5_ and mortality persists regardless of NO_2_ levels. However, NO_2_ appears to be protective against mortality at low levels when adjusted for PM_2.5_. We used a directed acyclic graph (DAG) to explore possible explanations [16] (Figure S3). If we assume there is no causal relationship between NO_2_ and mortality, and there exists a common pollution source. Conditioning, or adjusting for, PM_2.5_ should yield a null effect of NO_2_ on mortality. However, conditioning, or adjusting for, PM_2.5_ yielded an overall protective effect of NO_2_ and mortality; we hypothesize there may be a pollution source that produces NO_2_, but at the same time is an indicator of road traffic access or other indicators beneficial to health.

Although exposure to ambient nitrogen dioxide (NO_2_) has been linked to increased mortality in several epidemiological studies [6, 7], the question remains whether NO_2_ is directly responsible for the health effects or is only an indicator of other pollutants, including particulate matter. A systematic review using pooled data from Asia, North America, and Europe found evidence of long-term NO_2_ on mortality. They found greater similar risk estimates for total mortality of effects of PM_2.5_ than of NO_2_ for cardiovascular (20% versus 13%) and respiratory (5% versus 3%) mortality, per 10 μg/m^3^ of pollutants [6]. Another review got random-effects summary relative risks (RR) ranging from 1.02 to 1.06 for NO_2_ (per 10 μg/m^3^) and all-cause (24 cohorts), respiratory (15 cohorts), chronic obstructive pulmonary disease (COPD) (9 cohorts), and acute lower respiratory infection (5 cohorts) mortality. Meanwhile, it identified high levels of heterogeneity for all causes of death except COPD [7]. A time-series analysis using MCC data in 398 cities in 22 countries or regions found an association of NO_2_ and total cardiovascular and respiratory mortality [17]. The study used death records using ICD-9 or -10 codes. This study found the pooled concentration–response curves for all three causes were almost linear without discernible thresholds. A study in northern China also found higher NO_2_ was associated with lower all-cause mortality risk no matter adjusting or not adjusting for PM_10_ or SO_2_, and it only showed a harmful effect on lung cancer mortality when adjusting for PM_10_ [18]. Another study based on Dutch national databases found the positive association between NO_2_ and mortality remained for non-accidental and lung cancer mortality, but reversed for circulatory diseases mortality and disappeared for respiratory diseases mortality after adjusting for PM10 [19].

Our study has many strengths. First, we utilized a prospective cohort originally designed to ascertain determinants of healthy longevity, and thus our study benefited from having access to a wide range of confounders for adjustment. Our study’s sample size and various regions allowed us to see a wide spectrum of air pollution exposure levels, or having heterogenous exposures. Our study contains some limitations typical of observational epidemiologic studies and also specific to our study design. First, our exposure ascertainment of air pollutants relied in part on remote sensing modeling techniques, and we do not have a personalized air pollution monitor for each individual. Some people may live in households with biomass for cooking and heating and could suffer from high indoor air pollutants. Second, we cannot estimate cause-specific mortality because death was ascertained from next-of-kin, who could not report clinically accurate mortality causes. Third, there is potential unaccounted for residual confoundings, such as underlying social-economic statuses that lead to differential air pollution exposure and are related to the health outcome. Lastly, a possible exposure misclassification may arise NO_2_ may be a regional pollutant and could vary substantially in space, a difficulty for accurate exposure assessment. As the elderly population may spend most of their lives indoors, NO_2_’s penetration coefficients from outdoor to indoor are lower than PM_2.5_’s [20], which may have impacted our findings.

In the 2021 World Health Organization Air Quality Guidelines, the annual guideline for nitrogen dioxide (NO_2_) is four times tighter than the 2005 limit value and is down to 10 μg / m3, from 40 μg / m3. The 24-h guideline of 25 μg / m3 has been additionally introduced. PM_2.5_ threshold has been limited to a mere five μg / m3, from 10 µg per cubic meter (μg / m3). The 24-h average is 15 μg / m3, from 25 μg / m3. Both annual and 24-h average guidelines for PM10 are lower by five μg / m3 each. The US EPA integrated Science Assessment advises that NO_2_ is a suggested but not causal factor with mortality. The WHO global air quality guideline also is inconclusive on the causality of NO_2_ and mortality. Our study does not indicate an association of NO_2_ on mortality independent of PM_2.5_. It is possible that NO_2_ does not have a strong relationship with mortality at low levels, as there is evidence that the health effect of NO_2_ is on respiratory health only. Perhaps prior observed associations between ill health and NO_2_ at low concentrations in the ambient air result from co-exposure by particulate matter [21].

## Conclusion

Our findings indicate a consistent harmful effect of PM2.5 on all-cause mortality in a cohort of advanced aging population in China. We do not see harmful effects of NO_2_ when adjusted for PM_2.5_ on all-cause mortality. The results of our analysis suggest a complex interplay of these two air pollutants. We see consistent harmful effects of PM_2.5_ with all-cause pre-mature mortality among a cohort of elderly individuals. But NO_2_ was only harmful in some subgroups, namely colder regions. There are atmospheric explanations, such as the transfer of NO_2_ to PM_2.5_, exposure assessment situation where there is measurement error of the air pollution exposure, or that NO_2_ is more of a proxy for commercial activity, which in turn leads to better health, at least under the developing country context. Alternatively, it is possible that the NO_2_ does indeed have a null effect independent of PM_2.5_. Future studies of multiple pollutant models, along with temperature effect modification, are needed to determine the causal mechanisms for air pollution mixtures and health.

## Supplementary Information

**Additional file 1: Supplementary methods.** **Table S1.** Population Characteristics by High and Low PM2.5 and NO2 Exposure Levels (unit: μg/m^3^). **Table S2.** Association between NO2, PM2.5 levels and all-cause mortality adjusting for different variables. **Table S3.** The association between NO2, PM2.5 and mortality stratified by different exposure level groups (unit: μg/m^3^). **Figure S1.** The scatter plot of NO2 and PM2.5 of the year closest to outcome assessment. **Figure S2.** The restricted cubic spline of NO2 and PM2.5 on mortality. **Figure S3.** DAGs of NO2 and PM2.5 Relationship on Mortality.

## Data Availability

The CLHLS datasets are available from the Peking University Open Research Data (http://opendata.pku.edu.cn/dataverse/CHADS) and Inter-university Consortium at University of Michigan (https://www.icpsr.umich.edu/icpsrweb/NACDA/series/487).

## Abbreviations

NO_2_: Nitrogen dioxide
PM_2.5_: Fine particulate matter
CLHLS: Chinese Longitudinal Healthy Longevity Study
CRI: Cumulative Risk Index
HR: Hazard ratio
DAG: Directed acyclic graph
RR: Relative risks
COPD: Chronic obstructive pulmonary disease
