# Supplementary material for: NO2 and PM2.5 air pollution co-exposure and temperature effect modification on pre-mature mortality in advanced age: a longitudinal cohort study in China
Source: Environ Health. 2022 Oct 13;21:97. doi: 10.1186/s12940-022-00901-8 (PMC9559021; doi:10.1186/s12940-022-00901-8)
Supplement: Supplementary file 1 — Additional file 1: Supplementary methods. Table S1. Population Characteristics by High and Low PM2.5 and NO2 Exposure Levels (unit: μg/m3). Table S2. Association between NO2, PM2.5 levels and all-cause mortality adjusting for different variables. Table S3. The association between NO2, PM2.5 and mortality stratified by different exposure level groups (unit: μg/m3). Figure S1. The scatter plot of NO2 and PM2.5 of the year closest to outcome assessment. Figure S2. The restricted cubic spline of NO2 and PM2.5 on mortality. Figure S3. DAGs of NO2 and PM2.5 Relationship on Mortality. [file 12940_2022_901_MOESM1_ESM.docx]

**Supplementary materials**

**Supplementary methods**

We divided the regular exercise, smoking, and alcohol drinking status into three categories: “Current”, “Former”, and “Never”. For example, participants were asked, “do you do exercise regularly at present (planned exercise like walking, playing balls, running, and so on)?” and/or “did you do exercise regularly in the past?”. We defined the regular exercise status as “Current” for participants who answered “Yes” to the first question, “Former” for who answered “No” to the first question and “Yes” to the second question, and “Never” for who answered “No” to both two questions. Then we further quantified the current smoker based on the number of times smoked (or smoked) per day: <20 times/day and ≥20 times/day.

We defined those who drank equal or less than 14 g pure alcohol per day for the female or 28 g per day for the male as light drinkers, otherwise heavy drinkers. The unit of alcohol was a Chinese unit of weight called ‘Liang’ [50 grams (g)]. The level of alcohol consumption was calculated as drinks of alcohol per day, based on the beverage type and amount, assuming the following alcohol content by volume (v/v) typically seen in China: strong liquor 53%, weak liquor 38%, grape wine 12%, rice wine 15%, and beer 4% [1]. A standard drink was equal to 14.0 g of pure alcohol according to the criterion of the Center for Disease Control and Prevention in the USA, and moderate drinking is up to 1 drink per day for women and up to 2 drinks per day for men according to Dietary Guidelines for Americans 2015-2020.

1. Millwood IY, Li L, Smith M, Guo Y, Yang L, Bian Z, et al. Alcohol consumption in 0.5 million people from 10 diverse regions of china: Prevalence, patterns and socio-demographic and health-related correlates. Int J Epidemiol. 2013;42:816–27.

**Table S1. Population Characteristics by High and Low PM2.5 and NO2 Exposure Levels (unit: μg/m^3^)**

| **Variables** | **NO2<20 & PM2.5<35** | **NO2≥20 & PM2.5<35** | **NO2<20 & PM2.5≥35** | **NO2≥20 & PM2.5≥35** | **Overall** |
| --- | --- | --- | --- | --- | --- |
|  | **(N=1239)** | **(N=171)** | **(N=6490)** | **(N=3935)** | **(N=11835)** |
| **Age** |  |  |  |  |  |
| Mean (SD) | 82.0 (11.1) | 82.4 (11.4) | 87.4 (11.3) | 87.9 (11.4) | 86.9 (11.4) |
| Median [Min, Max] | 81.0 [65.0, 112] | 82.0 [65.0, 106] | 89.0 [65.0, 116] | 90.0 [65.0, 116] | 88.0 [65.0, 116] |
| **Gender** |  |  |  |  |  |
| Male | 564 (45.5) | 78 (45.6) | 2703 (41.6) | 1726 (43.9) | 5071 (42.8) |
| Female | 675 (54.5) | 93 (54.4) | 3787 (58.4) | 2209 (56.1) | 6764 (57.2) |
| **Education** |  |  |  |  |  |
| 0 year | 726 (58.6) | 79 (46.2) | 4350 (67.0) | 2314 (58.8) | 7469 (63.1) |
| 1-6 years | 405 (32.7) | 55 (32.2) | 1691 (26.1) | 1091 (27.7) | 3242 (27.4) |
| >6 years | 108 (8.7) | 37 (21.6) | 449 (6.9) | 530 (13.5) | 1124 (9.5) |
| **Marriage** |  |  |  |  |  |
| Married | 518 (41.8) | 80 (46.8) | 1895 (29.2) | 1216 (30.9) | 3709 (31.3) |
| not married | 721 (58.2) | 91 (53.2) | 4595 (70.8) | 2719 (69.1) | 8126 (68.7) |
| **Regular Exercise** |  |  |  |  |  |
| Current | 363 (29.3) | 90 (52.6) | 1456 (22.4) | 1253 (31.8) | 3162 (26.7) |
| Former | 160 (12.9) | 15 (8.8) | 646 (10.0) | 639 (16.2) | 1460 (12.3) |
| Never | 716 (57.8) | 66 (38.6) | 4388 (67.6) | 2043 (51.9) | 7213 (60.9) |
| **Smoking** |  |  |  |  |  |
| Never | 884 (71.3) | 123 (71.9) | 4344 (66.9) | 2495 (63.4) | 7846 (66.3) |
| Former | 164 (13.2) | 31 (18.1) | 950 (14.6) | 771 (19.6) | 1916 (16.2) |
| Light smoker | 122 (9.8) | 13 (7.6) | 938 (14.5) | 551 (14.0) | 1624 (13.7) |
| Heavy smoker | 69 (5.6) | 4 (2.3) | 258 (4.0) | 118 (3.0) | 449 (3.8) |
| **Drinking** |  |  |  |  |  |
| Never | 865 (69.8) | 112 (65.5) | 4456 (68.7) | 2741 (69.7) | 8174 (69.1) |
| Former | 171 (13.8) | 24 (14.0) | 882 (13.6) | 583 (14.8) | 1660 (14.0) |
| Light drinker | 66 (5.3) | 17 (9.9) | 419 (6.5) | 252 (6.4) | 754 (6.4) |
| Heavy drinker | 137 (11.1) | 18 (10.5) | 733 (11.3) | 359 (9.1) | 1247 (10.5) |
| **Household Income** |  |  |  |  |  |
| <4000 | 382 (30.8) | 13 (7.6) | 1755 (27.0) | 591 (15.0) | 2741 (23.2) |
| <10000 | 306 (24.7) | 33 (19.3) | 1652 (25.5) | 664 (16.9) | 2655 (22.4) |
| <20000 | 256 (20.7) | 41 (24.0) | 1469 (22.6) | 887 (22.5) | 2653 (22.4) |
| ≥20000 | 295 (23.8) | 84 (49.1) | 1614 (24.9) | 1793 (45.6) | 3786 (32.0) |
| **BMI Groups** |  |  |  |  |  |
| <18.5 | 512 (41.3) | 34 (19.9) | 2284 (35.2) | 1063 (27.0) | 3893 (32.9) |
| <25 | 667 (53.8) | 111 (64.9) | 3721 (57.3) | 2396 (60.9) | 6895 (58.3) |
| <30 | 54 (4.4) | 22 (12.9) | 420 (6.5) | 401 (10.2) | 897 (7.6) |
| ≥30 | 6 (0.5) | 4 (2.3) | 65 (1.0) | 75 (1.9) | 150 (1.3) |
| **Residence** |  |  |  |  |  |
| Urban | 360 (29.1) | 119 (69.6) | 1665 (25.7) | 2160 (54.9) | 4304 (36.4) |
| Rural | 879 (70.9) | 52 (30.4) | 4825 (74.3) | 1775 (45.1) | 7531 (63.6) |
| **Geographical Region within China** |  |  |  |  |  |
| Central | 102 (8.2) | 4 (2.3) | 2110 (32.5) | 758 (19.3) | 2974 (25.1) |
| Eastern | 273 (22.0) | 68 (39.8) | 1488 (22.9) | 1558 (39.6) | 3387 (28.6) |
| Northeastern | 101 (8.2) | 62 (36.3) | 208 (3.2) | 534 (13.6) | 905 (7.6) |
| Northern | 19 (1.5) | 0 (0) | 143 (2.2) | 486 (12.4) | 648 (5.5) |
| Southern | 699 (56.4) | 33 (19.3) | 1452 (22.4) | 206 (5.2) | 2390 (20.2) |
| Southwestern | 45 (3.6) | 4 (2.3) | 1089 (16.8) | 393 (10.0) | 1531 (12.9) |

**Table S2. Association between NO2, PM2.5 levels and all-cause mortality adjusting for different variables**

| **Adjustment variables** | **NO2 (μg/m^3^)** | | |  | **PM2.5 (μg/m^3^)** | | |
| --- | --- | --- | --- | --- | --- | --- | --- |
|  | **[ 40.0,109.0] as reference (n=868)** | **HR (95% CI)** | **p value** |  | **[ 70.0,133.1] as reference (n=1663)** | **HR (95% CI)** | **p value** |
| **Age, gender** | [1.0, 10.0) (n=2807) | 0.924 (0.84, 1.017) | 0.108 |  | [14.8, 25.0) (n=222) | 0.302 (0.243, 0.376) | <0.001 |
|  | [10.0, 20.0)  (n=4922) | 0.968 (0.883, 1.06) | 0.483 |  | [25.0, 35.0) (n=1188) | 0.3 (0.27, 0.333) | <0.001 |
|  | [20.0, 30.0) (n=2374) | 1.161 (1.054, 1.279) | 0.002 |  | [35.0, 50.0) (n=4131) | 0.632 (0.592, 0.676) | <0.001 |
|  | [30.0, 40.0) (n=864) | 1.123 (0.999, 1.262) | 0.052 |  | [50.0, 70.0) (n=4631) | 0.798 (0.75, 0.85) | <0.001 |
| **Age, gender, education, household income** | [1.0, 10.0) | 0.886 (0.802, 0.979) | 0.017 |  | [14.8, 25.0) | 0.3 (0.241, 0.373) | <0.001 |
|  | [10.0, 20.0) | 0.927 (0.843, 1.019) | 0.118 |  | [25.0, 35.0) | 0.299 (0.269, 0.332) | <0.001 |
|  | [20.0, 30.0) | 1.12 (1.015, 1.236) | 0.024 |  | [35.0, 50.0) | 0.631 (0.591, 0.674) | <0.001 |
|  | [30.0, 40.0) | 1.101 (0.979, 1.238) | 0.109 |  | [50.0, 70.0) | 0.796 (0.748, 0.847) | <0.001 |
| **Age, gender, education, household income, marital status, smoking status, drinking status, physical activity, residence** | [1.0, 10.0) | 0.815 (0.733, 0.906) | <0.001 |  | [14.8, 25.0) | 0.309 (0.248, 0.385) | <0.001 |
|  | [10.0, 20.0) | 0.867 (0.785, 0.958) | 0.005 |  | [25.0, 35.0) | 0.299 (0.269, 0.332) | <0.001 |
|  | [20.0, 30.0) | 1.056 (0.954, 1.169) | 0.295 |  | [35.0, 50.0) | 0.632 (0.592, 0.676) | <0.001 |
|  | [30.0, 40.0) | 1.054 (0.936, 1.186) | 0.385 |  | [50.0, 70.0) | 0.799 (0.751, 0.851) | <0.001 |
| **Age, gender, education, household income, marital status, smoking status, drinking status, physical activity, ﻿ residence, geographical region of residence.** | [1.0, 10.0) | 0.794 (0.707, 0.892) | <0.001 |  | [14.8, 25.0) | 0.206 (0.164, 0.259) | <0.001 |
|  | [10.0, 20.0) | 0.857 (0.77, 0.953) | 0.004 |  | [25.0, 35.0) | 0.219 (0.195, 0.246) | <0.001 |
|  | [20.0, 30.0) | 1.055 (0.948, 1.173) | 0.326 |  | [35.0, 50.0) | 0.506 (0.469, 0.547) | <0.001 |
|  | [30.0, 40.0) | 1.059 (0.939, 1.196) | 0.349 |  | [50.0, 70.0) | 0.724 (0.677, 0.773) | <0.001 |
| **Age, gender, education, household income, marital status, smoking status, drinking status, physical activity, ﻿ residence, geographical region of residence, BMI** | [1.0, 10.0) | 0.79 (0.703, 0.887) | <0.001 |  | [14.8, 25.0) | 0.202 (0.161, 0.254) | <0.001 |
|  | [10.0, 20.0) | 0.853 (0.767, 0.949) | 0.003 |  | [25.0, 35.0) | 0.215 (0.192, 0.242) | <0.001 |
|  | [20.0, 30.0) | 1.057 (0.95, 1.175) | 0.308 |  | [35.0, 50.0) | 0.502 (0.465, 0.542) | <0.001 |
|  | [30.0, 40.0) | 1.063 (0.942, 1.199) | 0.325 |  | [50.0, 70.0) | 0.719 (0.673, 0.768) | <0.001 |

**Table S3. The association between NO2, PM2.5 and mortality stratified by different exposure level groups (unit: μg/m^3^)**

| **Groups** | **Exposure** | **n** | **Single pollutant model-NO2** | |  | | **Single pollutant model-PM2.5** | | |  | **Two pollutants model - NO2+PM2.5** | |
| --- | --- | --- | --- | --- | --- | --- | --- | --- | --- | --- | --- | --- |
|  |  |  | **HR (95% CI)** | **p value** | |  | | **HR (95% CI)** | **p value** |  | **HR (95% CI)** | **p value** |
| **NO2<16 & PM2.5<51** | **NO2** | 3820 | 0.956 (0.86, 1.064) | 0.411 | |  | |  |  |  | 0.695 (0.62, 0.779) | <0.001 |
|  | **PM2.5** | 3820 |  |  | |  | | 2.037 (1.907, 2.175) | <0.001 |  | 2.135 (1.996, 2.284) | <0.002 |
|  |  |  |  |  | |  | |  |  |  |  |  |
|  | NO2 t1 [ 1.22, 6.37) | 1274 | Ref | / | |  | |  |  |  | Ref | / |
|  | NO2_t2 [ 6.37,11.34) | 1273 | 0.913 (0.823, 1.012) | 0.082 | |  | |  |  |  | 0.835 (0.752, 0.927) | 0.001 |
|  | NO2 t3 [11.34,15.99] | 1273 | 0.949 (0.852, 1.058) | 0.345 | |  | |  |  |  | 0.751 (0.671, 0.841) | <0.001 |
|  | PM2.5 t1﻿ [14.8,36.0) | 1280 |  |  | |  | | Ref | / |  | Ref | / |
|  | PM2.5 t2 [36.0,42.6) | 1269 |  |  | |  | | 2.159 (1.926, 2.42) | <0.001 |  | 2.237 (1.994, 2.51) | <0.001 |
|  | PM2.5 t3 [42.6,50.9] | 1271 |  |  | |  | | 3.351 (2.988, 3.758) | <0.001 |  | 3.521 (3.134, 3.956) | <0.001 |
|  |  |  |  |  | |  | |  |  |  |  |  |
| **NO2≥16 & PM2.5<51** | **NO2** | 2007 | 1.047 (1.007, 1.088) | 0.020 | |  | |  |  |  | 1.035 (0.996, 1.076) | <0.003 |
|  | **PM2.5** | 2007 |  |  | |  | | 1.563 (1.41, 1.733) | <0.001 |  | 1.555 (1.402, 1.724) | <0.004 |
|  |  |  |  |  | |  | |  |  |  |  |  |
|  | NO2 t1 [16, 20) | 669 | Ref | / | |  | |  |  |  | Ref | / |
|  | NO2 t2 [20, 27) | 669 | 0.929 (0.809, 1.068) | 0.300 | |  | |  |  |  | 0.935 (0.813, 1.075) | 0.344 |
|  | NO2 t3 [27,109] | 669 | 1.27 (1.092, 1.477) | 0.002 | |  | |  |  |  | 1.197 (1.029, 1.394) | 0.020 |
|  | PM2.5 t1 ﻿[18.7,41.2) | 675 |  |  | |  | | Ref | / |  | Ref | / |
|  | PM2.5 t2 [41.2,46.6) | 668 |  |  | |  | | 1.584 (1.363, 1.841) | <0.001 |  | 1.542 (1.326, 1.794) | <0.001 |
|  | PM2.5 t3﻿ [46.6,50.9] | 664 |  |  | |  | | 2.048 (1.766, 2.376) | <0.001 |  | 2.003 (1.726, 2.326) | <0.001 |
|  |  |  |  |  | |  | |  |  |  |  |  |
| **NO2<16 & PM2.5≥51** | **NO2** | 2094 | 0.751 (0.642, 0.88) | <0.001 | |  | |  |  |  | 0.706 (0.601, 0.829) | <0.005 |
|  | **PM2.5** | 2094 |  |  | |  | | 1.19 (1.107, 1.28) | <0.001 |  | 1.217 (1.132, 1.309) | <0.006 |
|  |  |  |  |  | |  | |  |  |  |  |  |
|  | NO2 t1 [ 2.37,10.4) | 698 | Ref | / | |  | |  |  |  | Ref | / |
|  | NO2 t2 [10.36,13.4) | 698 | 0.736 (0.65, 0.834) | <0.001 | |  | |  |  |  | 0.737 (0.649, 0.836) | <0.001 |
|  | NO2 t3 [13.36,16.0] | 698 | 0.827 (0.73, 0.937) | 0.003 | |  | |  |  |  | 0.805 (0.71, 0.914) | 0.001 |
|  | PM2.5 t1﻿ [51.0,57.1) | 702 |  |  | |  | | Ref | / |  | Ref | / |
|  | PM2.5 t2﻿ [57.1,63.7) | 703 |  |  | |  | | 0.915 (0.802, 1.044) | 0.186 |  | 0.96 (0.841, 1.096) | 0.547 |
|  | PM2.5 t3﻿ [63.7,87.7] | 689 |  |  | |  | | 1.321 (1.158, 1.508) | <0.001 |  | 1.368 (1.198, 1.562) | <0.001 |
|  |  |  |  |  | |  | |  |  |  |  |  |
| **NO2≥16 & PM2.5≥51** | **NO2** | 3914 | 1.038 (0.998, 1.079) | 0.061 | |  | |  |  |  | 1.028 (0.987, 1.07) | <0.007 |
|  | **PM2.5** | 3914 |  |  | |  | | 1.042 (1.008, 1.077) | 0.014 |  | 1.037 (1.002, 1.072) | <0.008 |
|  |  |  |  |  | |  | |  |  |  |  |  |
|  | NO2 t1 ﻿[16.0, 20.5) | 1305 | Ref | / | |  | |  |  |  | Ref | / |
|  | NO2 t2 [20.5, 28.4) | 1305 | 1.1 (1.008, 1.2) | 0.032 | |  | |  |  |  | 1.081 (0.99, 1.18) | 0.081 |
|  | NO2 t3 [28.4,106.6] | 1304 | 1.051 (0.945, 1.169) | 0.356 | |  | |  |  |  | 1.002 (0.899, 1.116) | 0.977 |
|  | PM2.5 t1﻿ [51.0, 60.0) | 1309 |  |  | |  | | Ref | / |  | Ref | / |
|  | PM2.5 t2 [60.0, 70.4) | 1309 |  |  | |  | | 0.953 (0.866, 1.047) | 0.315 |  | 0.953 (0.867, 1.048) | 0.322 |
|  | PM2.5 t3 [70.4,133.1] | 1296 |  |  | |  | | 1.231 (1.115, 1.36) | <0.001 |  | 1.232 (1.114, 1.364) | <0.001 |

Note: NO2 and PM2.5 were the annual average value of the year closest to outcome assessment; All models adjusted for age, gender, education, household income, marital status, smoking status, drinking status, physical activity, residence, geographical region of residence, and BMI.

**
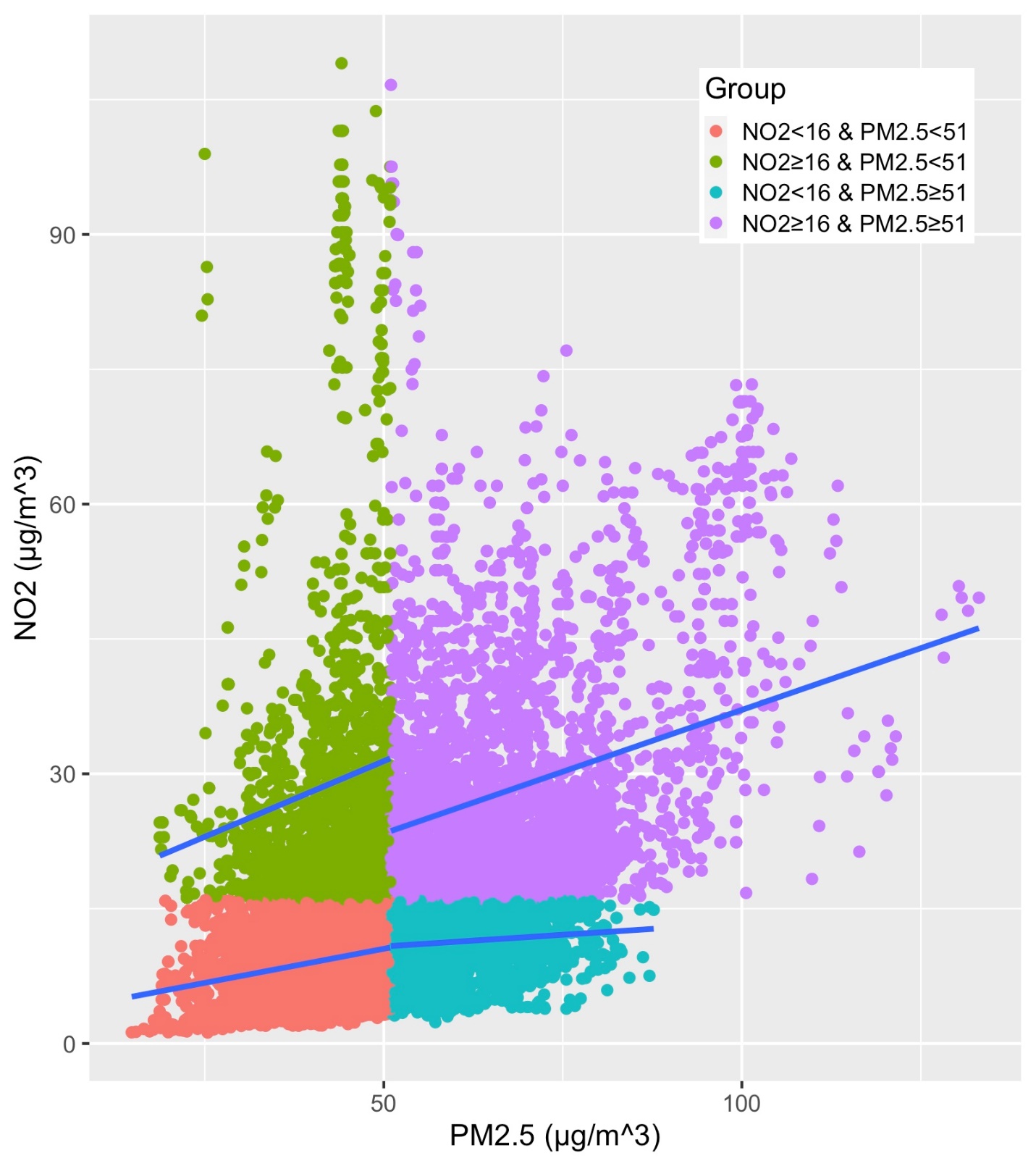
**

**Figure S1. The scatter plot of NO2 and PM2.5 of the year closest to outcome assessment**

**
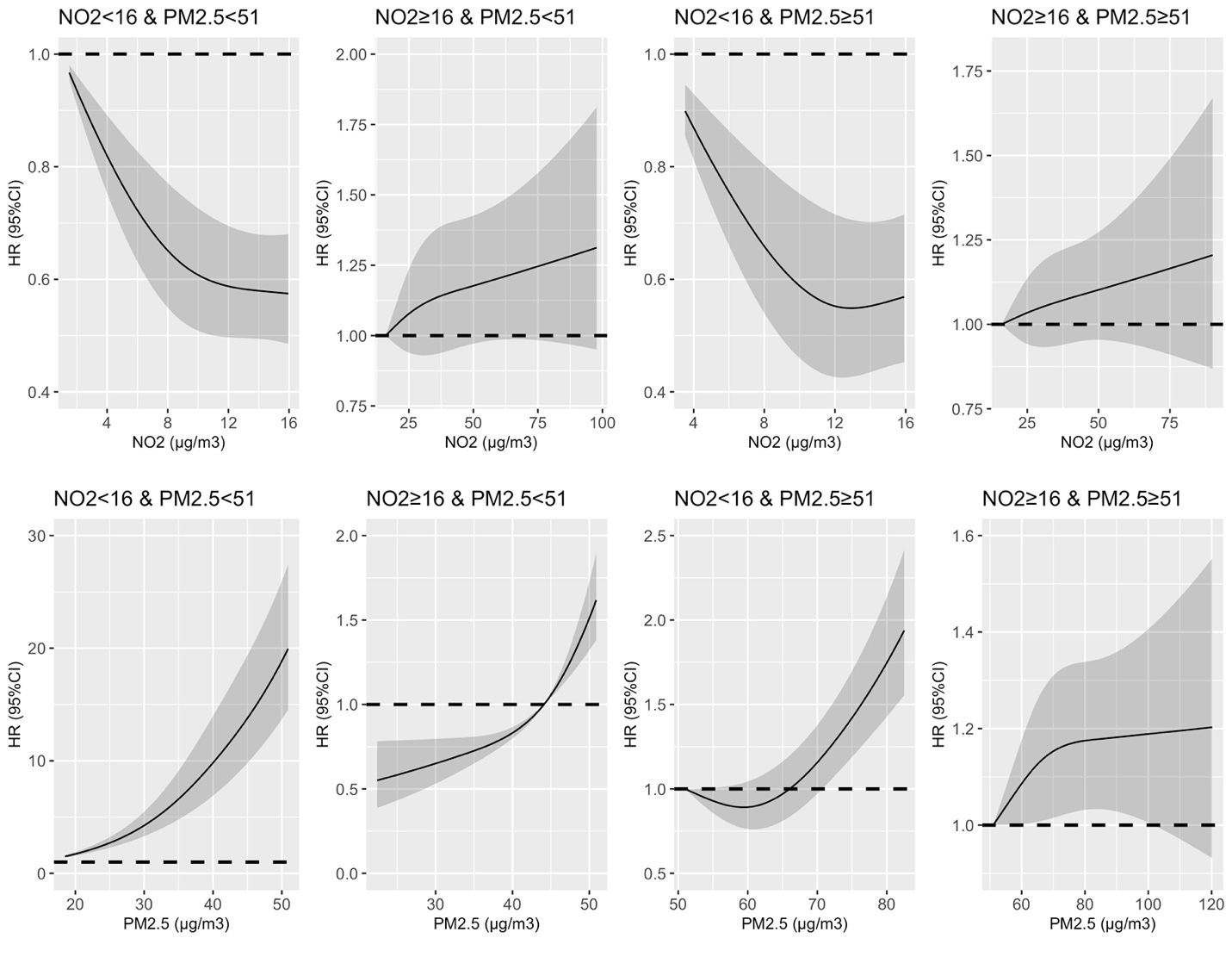
**

**Figure S2. The restricted cubic spline of NO2 and PM2.5 on mortality**

Note: NO2 and PM2.5 were the annual average value of the year closest to outcome assessment. All models adjusted for age, gender, education, household income, marital status, smoking status, drinking status, physical activity, residence, geographical region of residence, and BMI. Models for NO2 additionally adjusted for PM2.5 and models for PM2.5 additionally adjusted for NO2.

**
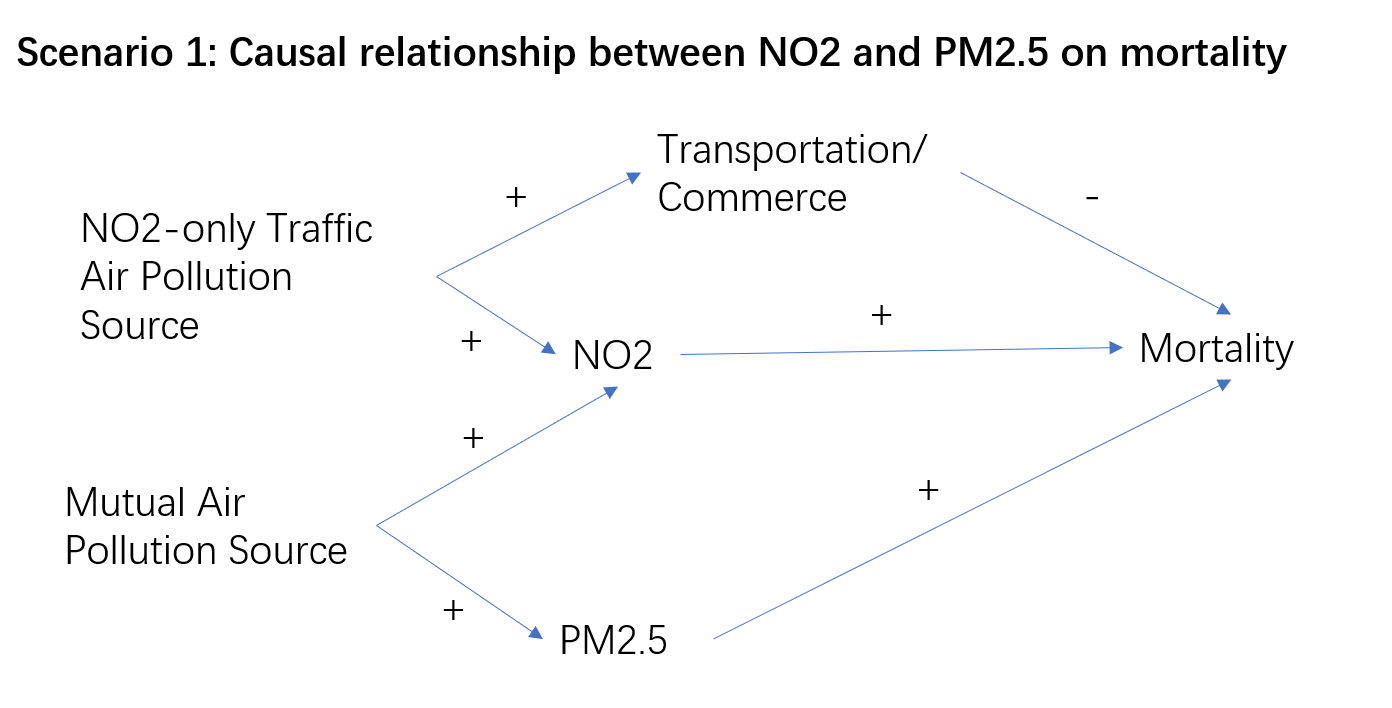
**

**
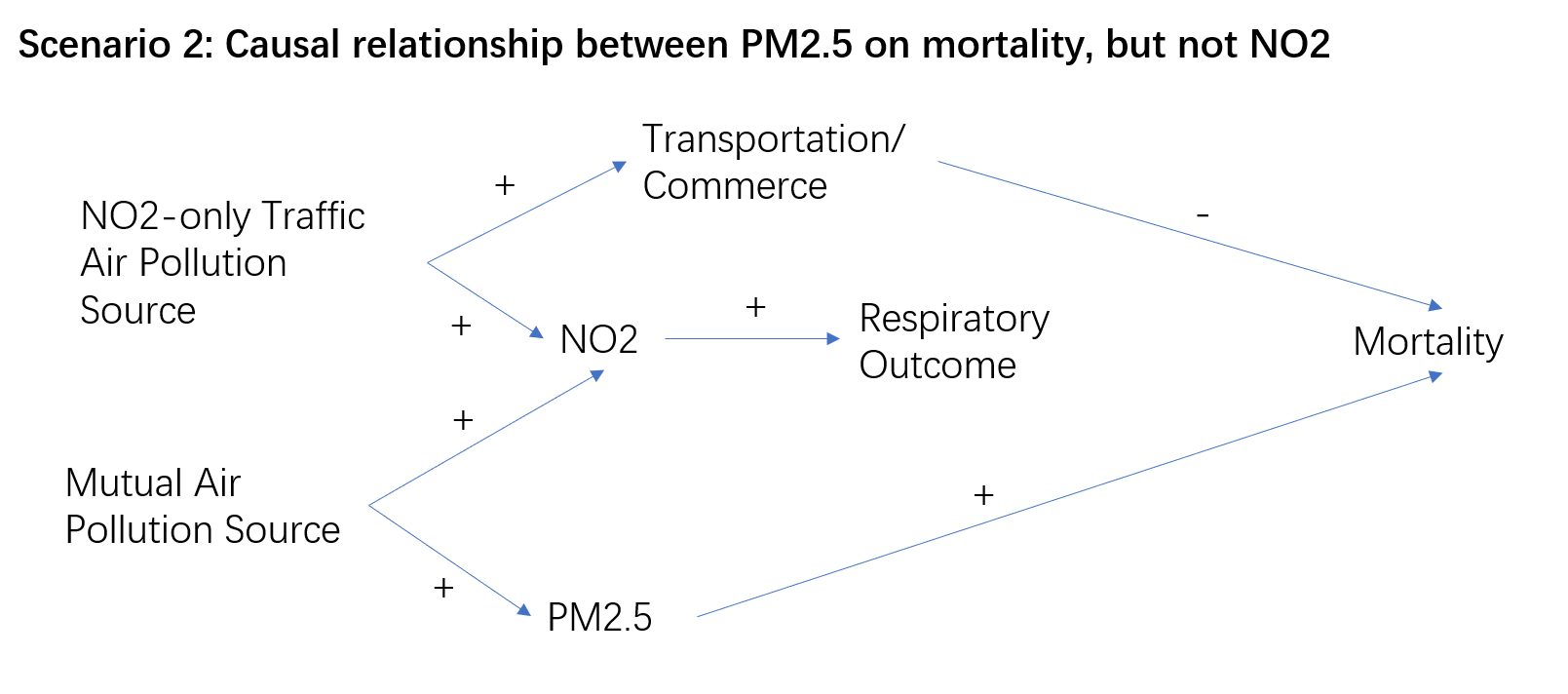
** **
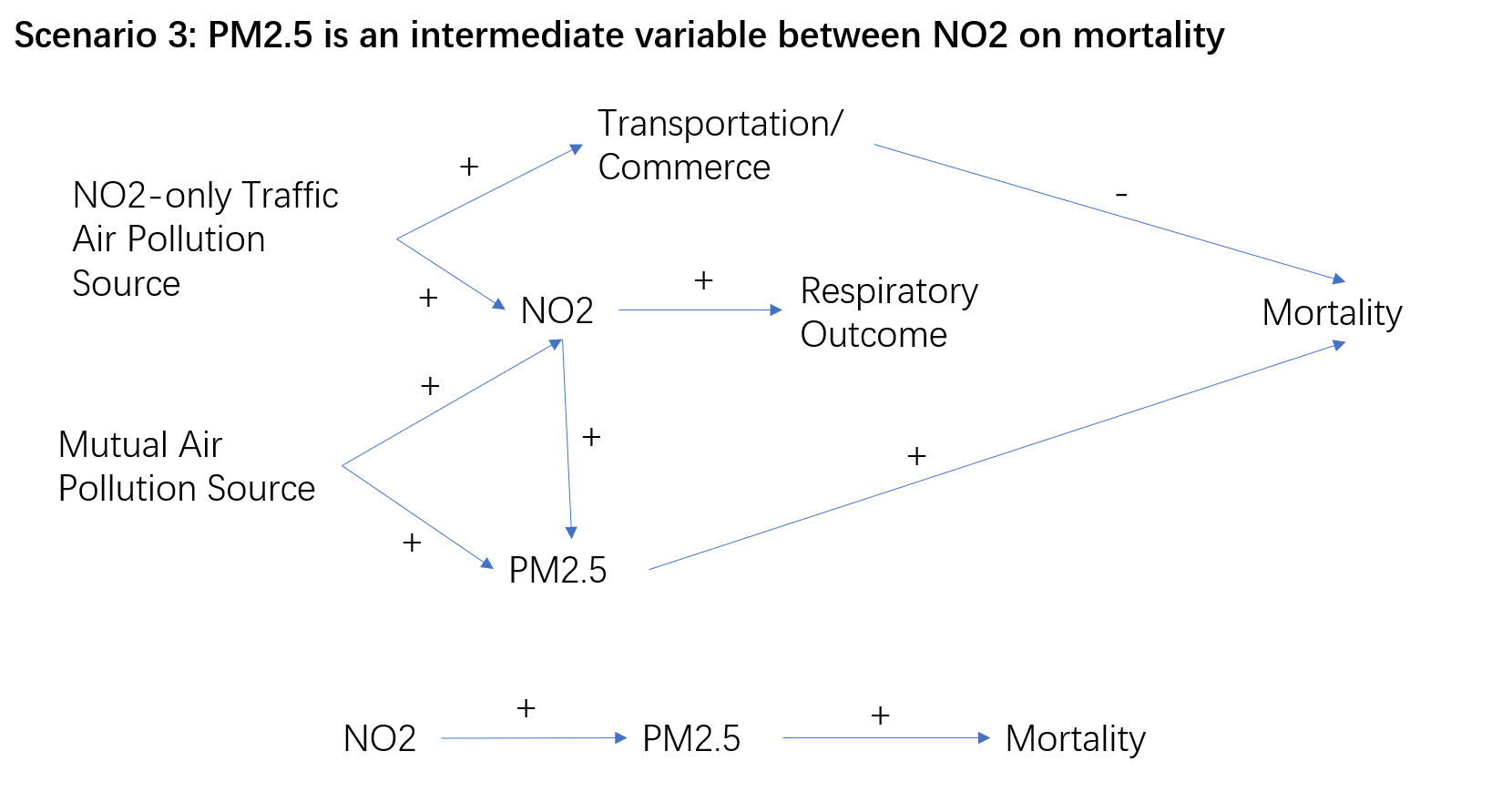
**

**Figure S3. DAGs of NO2 and PM2.5 Relationship on Mortality**
